# Theoretical insights into excited-state hydrogen bonding effects and intramolecular proton transfer (ESIPT) mechanism for BTS system

**DOI:** 10.1038/s41598-020-61804-7

**Published:** 2020-03-20

**Authors:** Jiemin Wang, Qiang Liu, Dapeng Yang

**Affiliations:** 1 Department of Physics & Electronic Information, Luoyang Normal University, Luoyang, 471934 P.R. China; 2 Henan Key laboratory of Electromagnetic Transformation and Detection, Luoyang, 471934 P.R. China; 3 State Key Laboratory of Molecular Reaction Dynamics, Dalian Institute of Chemical Physics, Chinese Academy of Sciences, Dalian, 116023 P.R. China

**Keywords:** Single-molecule fluorescence, Electronic devices

## Abstract

In this work, N,N’-bis(salicylidene)-(2-(3′,4′-diaminophenyl)benzothiazole) (named as “BTS”) system was studied about its excited-state intramolecular proton transfer (ESIPT) process. The analyses about reduced density gradient (RDG) reveal the formation of two intramolecular hydrogen bonds in BTS system. Bond lengths and angles, infrared (IR) vibrations as well as frontier molecular orbitals (MOs) using TDDFT method indicate that the strength of hydrogen bond should be enhanced in the S_1_ state. Particularly, hydrogen bond O1–H2···N3 undergoes larger variations compared with O4–H5···N6, which infers that hydrogen bond O1–H2···N3 may play a decisive role in the ESIPT process of BTS system. Given the two hydrogen bonds of BTS molecule, two types of potential energy curves have been constructed, which confirms that only single proton transfer process occurs due to lower energy barrier along with O1–H2···N3 rather than O4–H5···N6. This work not only presents a reasonable explanation for previous experiment, but also clarifies the specific ESIPT mechanism for BTS system.

## Introduction

As one of the most fundamental weak interaction, intra- as well as inter- molecular hydrogen bond is illocal in nature^1–3^. Proton transfer (PT), as the elementary class of photochemical and photophysical domains happening along with pre-existing hydrogen bond, has attracted much attention during the last few decades^4–8^. Upon photoexcitation, excited-state intra- (inter-) molecular proton transfer (ESIPT) process is the initial step of many photobiological and photochemical reactions, which is crucial in nature. Due to the transient properties, ESIPT processes have been adopted in several applications recently including molecular logic gates, UV filters, fluorescence sensors, etc.^9–18^. By the light of nature, both applied and cognitive attention have been paid to ESIPT phenomenon, which becomes a demanding subject of research^19–25^.

Generally, ESIPT means the transfer of a hydroxyl (or amino) proton to an oxygen (or nitrogen) acceptor via pre-existing hydrogen bonds. Upon photoexcitation, an unstable position of proton is resulted from the projection of the nuclear wave function of molecule on the excited-state potential energy surface (PES)^26–35^. The driving force for ESIPT is provided by the energy gap between the initial and relaxed excited states. Demonstrating by the mirror symmetry between absorption and emission spectra, the nuclear configuration of the target molecule remains close to that of the ground state over the excited-state lifetime. The mirror symmetry should be broken up by the influence of ESIPT on the Franck-Condon factors^26–35^. The proton-transfer tautomer emits fluorescence at longer wavelength and results in larger Stokes shifts.

**Figure 1:**
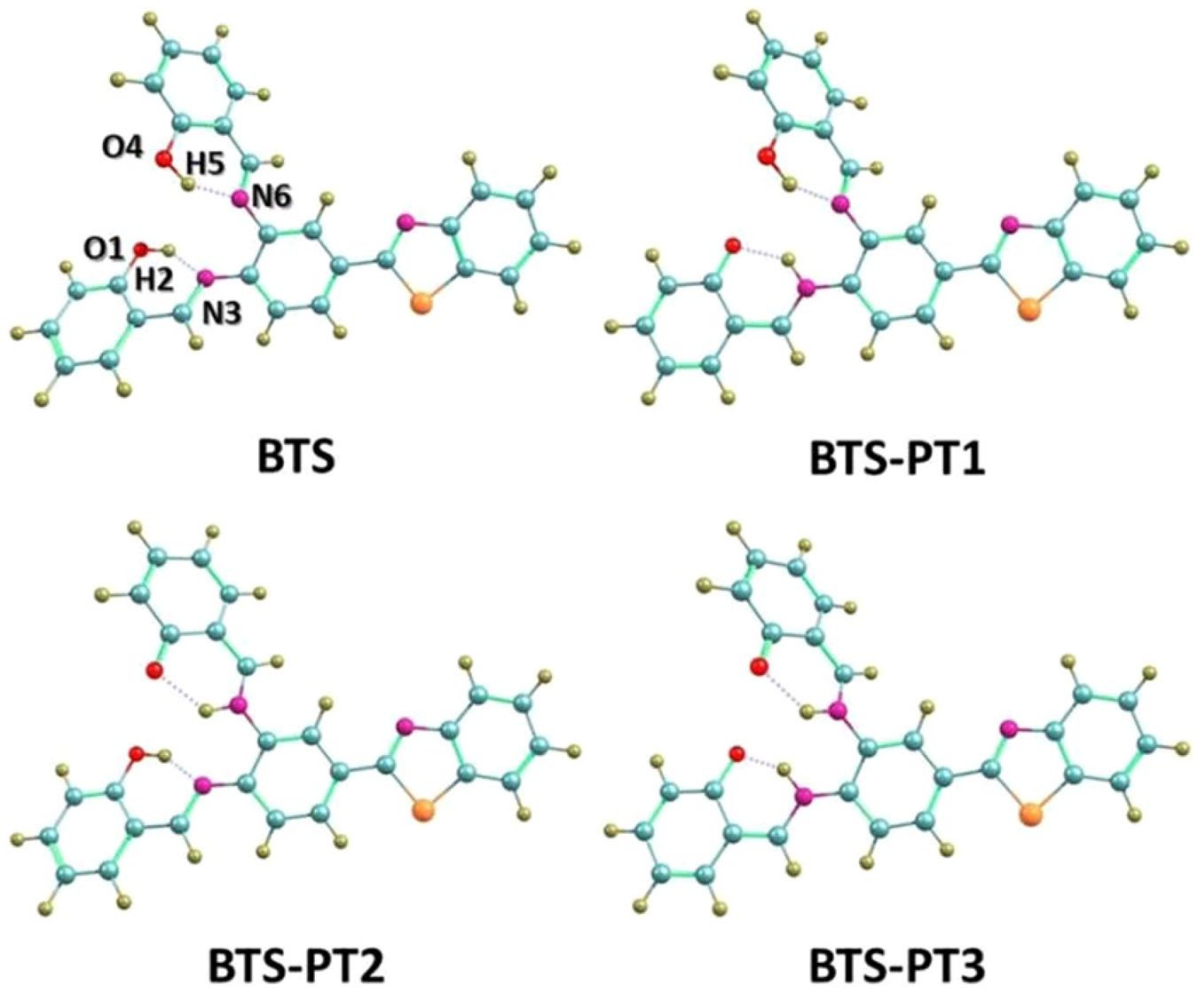
View of the relative structures for BTS, BTS-PT1, BTS-PT2 and BTS-PT3.

As far as we know, single ESPT process may not be sufficient to investigate the complex hydrogen bonding behaviors in biological fields, since more and more photo-induced mutations refer to multiple protons^36–39^. Just because of this reason, more and more people pay attention to the ESIPT reaction involved in double hydrogen bonding wires, since this kind of ESIPT process is the most fundamental case and precondition for further exploring multiple PT behaviors. Recently, Duarte and co-workers reported a novel N,N’-bis(salicylidene)-(2-(3′, 4′-diaminophenyl)benzothiazole) (named as “BTS”) system^40^, in which dual hydrogen bonds are formed, as shown in Fig. 1. BTS was successfully tested as the active layer in organic white light emitting diodes^40^. The larger Stokes shift experimentally observed by Duarte and co-workers indicates that ESIPT may happen and modulate the excited-state dynamical process of BTS^40^. Since BTS owns two intramolecular hydrogen bonds, it is possible for the molecule to undergo ESIPT process along with one or two hydrogen bonds. However, in this previous work^40^, Duarte and co-workers did not pay much attention to how the ESIPT process takes place in S_1_ state and whether single or double proton transfer process happens is not clear up to now. If the double protons transfer is inhibited, which hydrogen bond is the ESIPT path? In order to offer a detailed and clear excited-state dynamical process for BTS system, theoretical investigation on BTS has been carried out with DFT and TDDFT methods^41–44^ in the present work. The TDDFT method has been recognized as a quite useful tool in the theoretical research of hydrogen-bonded systems^45–52^.

## Theoretical methods

In the present work, all theoretical calculations presented have been performed based on the DFT and TDDFT methods with Becke’s three-parameter hybrid exchange function with the Lee-Yang-Parr gradient-corrected correlation functional (B3LYP)^53–55^ as well as the triple-ζ valence quality with one set of polarisation functions (TZVP)^56^ basis set by Gaussian 09 programs^57^. Because the experiment were conducted in DCM solvent, in all calculations, the solvent effect (DCM) was selected based on the Polarizable Continuum Model (PCM) using the integral equation formalism variant (IEF-PCM)^58–60^. The geometries of S0 and S1 states for all the relative structures were optimized without constrain of bonds, angles and dihedral angles. Vibrational frequency calculations have been used to analyze the optimized structures to confirm that these structures corresponded to the local minima on the S0 and S1 PESs (no imaginary frequency). The calculations of vertical excitation energies were also performed from the ground-optimized structures based on TDDFT methodology with IEF-PCM, and our theoretical calculations predicted the six low-lying absorbing transitions. The S0 and S1 potential energy curves of the BTS system have been scanned by constraining optimizations and frequency analyses to obtain the thermodynamic corrections in the corresponding electronic state. Harmonic vibrational frequencies in the ground and excited state were determined by diagonalization of the Hessian. The excited-state Hessian was obtained by numerical differentiation of the analytical gradients using central differences and default displacements of 0.02 Bohr. The infrared intensities were determined from the gradients of the dipole moment.

## Results and discussion

**Figure 2:**
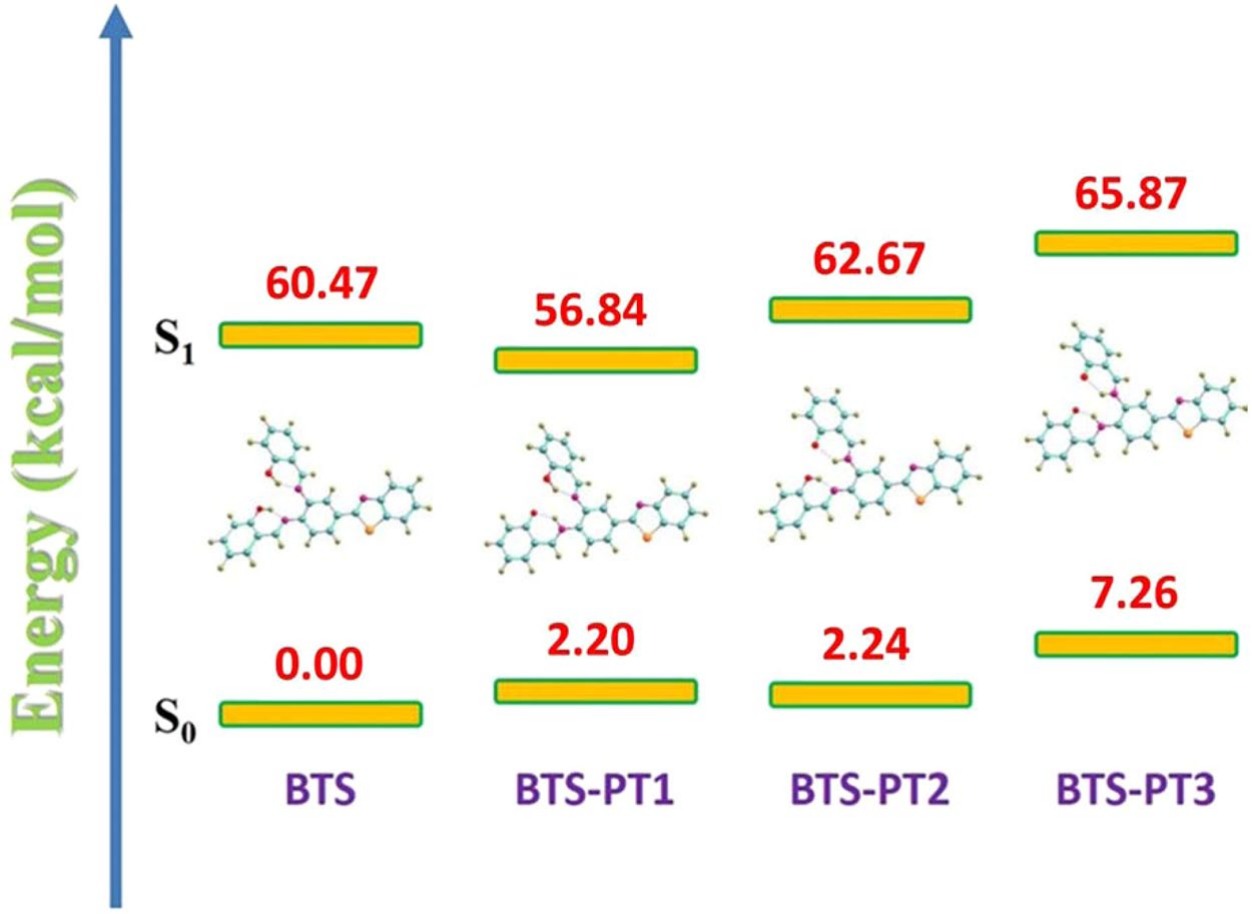
The energy diagram presents energy gaps among the four stable structures (i.e., BTS, BTS-PT1, BTS-PT2 and BTS-PT3) in both S_0_ and S_1_ states. Herein, the optimized S_0_-state BTS is selected as the energy zero point.

At B3LYP/TZVP theoretical level, the structural optimizations of the four molecular configurations were performed with DFT/TDDFT methods. The analyses of vibrational frequency were carried out so that the stationary properties of the relative structures can be insured. The structures of BTS, BTS-PT1 (one proton transfer via O1–H2···N3), BTS-PT2 (one proton transfer via O4–H5···N6) and BTS-PT3 (double proton transfer structure) are displayed in Fig. 1. Figure 2 shows the energy relationships of these four stable configurations in both ground and first excited states. Firstly, we have confirmed the formation of two intramolecular hydrogen bonds in BTS molecule and shown the results in Figure S1. According to previous report by Johnson *et al*.^61^, the spikes located around −0.02 a.u. in Figure S1 reveal the hydrogen bonding effects in real space. Based on the atom in molecule (AIM) theory, to assign an interaction between two primary atoms, the necessary and sufficient conditions are the identification of a critical point (CP) and the existence of the bond path in equilibrium geometry. It is shown by the relative AIM topologic parameters that the *ρ*(*r*) at the CP moiety for both hydrogen bonds are close to 0.04 a.u., which is the maximum threshold value ensuring the formation of hydrogen bond^62–64^. Furthermore, the relative ∇^2^ρ_c_ values of them are in the range of 0.02–0.15 a.u^62–64^. Hence, there are reasons to believe that two hydrogen bonds are formed in the ground-state BTS.

To inspect the changes about geometrical structures, some primary parameters of the two hydrogen bonds in BTS molecule are listed in Table 1. Upon photoexcitation, O1–H2 is elongated from 0.998 Å (S_0_) to 1.003 Å (S_1_), which undergoes a relatively large change compared with O4–H5 (i.e. from 0.998 Å to 0.999 Å). It indicates that hydrogen bond O1–H2···N3 will be largely affected by photoexcitation. As far as we know, the hydrogen bond length involved in ESIPT moieties is important, since both the acidity of proton donor and the basicity of proton acceptor could change upon photoexcitation. As shown in Table 1, the distance between H2···N3 is shortened from 1.722 (S_0_) to 1.693 Å (S_1_), which reveals the stronger attraction of proton acceptor N3 moiety upon photoexcitation to the first excited singlet state. In view of the bond angle for BTS, it should be noted that δ(O1–H2···N3) increases from 147.4° in ground state to 148.8° in first excited state whereas δ(O4–H5···N6) decreases slightly (147.5°→147.1°). Thus, we can predict that O1–H2···N3 will be strengthened in the S_1_ state^45–52^.

**Table 1:** Bond lengths (Å) and bond angles (°) of BTS, BTS-PT1 and BTS-PT2 forms involved in hydrogen bonds in both S_0_ and S_1_ states.

	BTS		BTS-PT1		BTS-PT2	
	S_0_	S_1_	S_0_	S_1_	S_0_	S_1_
O1–H2	0.998	1.003	1.756	1.812	0.992	0.996
H2–N3	1.722	1.693	1.036	1.035	1.762	1.734
O4–H5	0.998	0.999	0.992	0.992	1.755	1.892
H5–N6	1.719	1.721	1.770	1.766	1.036	1.030
δ(O1H2N3)	147.4	148.8	137.2	137.4	146.6	150.2
δ(O4H5N6)	147.5	147.1	146.5	146.8	137.3	132.4

**Figure 3:**
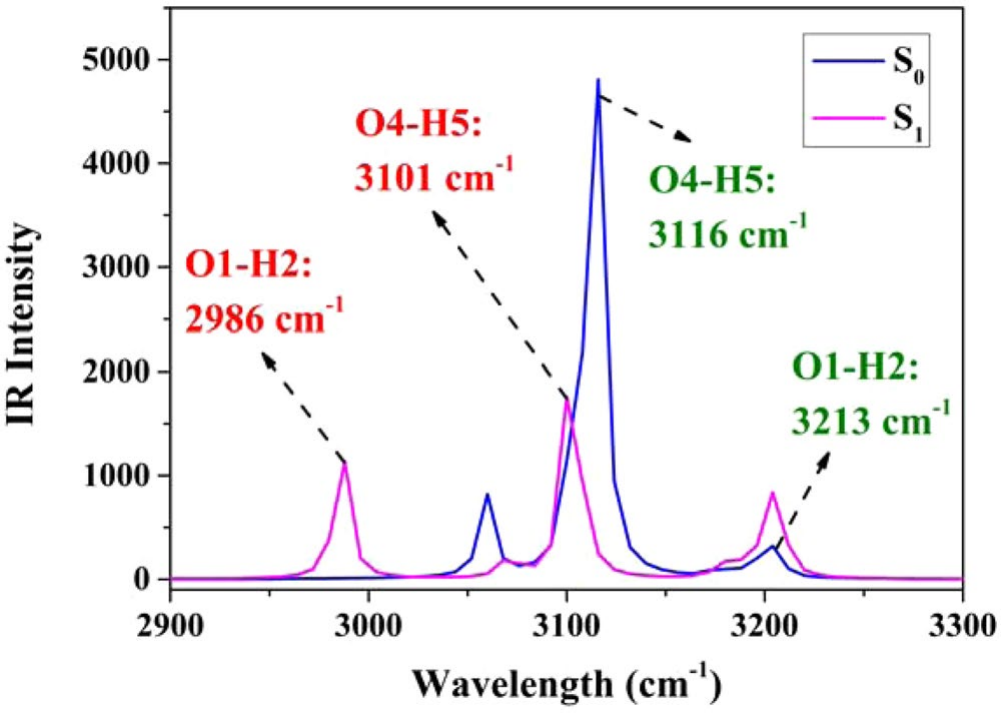
The theoretical IR spectra of the O1–H2 and O4–H5 stretching vibrational modes for BTS molecule in both S_0_ and S_1_ states.

As another improtant index for investigating hydrogen bond^45–52^, infrared (IR) vibrational spectral shifts have also been adopted to uncover the effects of photoexcitation (seen in Fig. 3). Obviously, the theoretical stretching vibration of O4–H5 indicates a small 15 cm^−1^ red shift (i.e., 3116 cm^−1^ (S_0_) → 3101 cm^−1^ (S_1_)). Contrarily, a larger red shift 227 cm^−1^ for O1–H2 could be formed upon photoexcitation (3213 cm^−1^ (S_0_) → 2986 cm^−1^ (S_1_)). All these variations indicate that hydrogen bond O1–H2···N3 will be strengthened more than O4–H5···N6, which should provide the possibility for ESIPT process only via the former. In addition, to provide the most conspicuous evidence of the strengthening of the two hydrogen bonds upon photoexcitation to the first singlet excited state S_1_, we have adopted the topological analyses to calculate the hydrogen bonding energies (E_HB_)^65^. As mentioned by Espinosa *et al*.^65^, the relationship between potential energy density V(r) and E_HB_ at corresponding BCP (AIM analyses) could be written as: E_HB_ = V(r_BCP_)/2. Therefore, the ground-state E_HB_ of O1–H2···N3 and O4–H5···N6 are 7.59 and 7.39 kcal/mol, respectively. In the first excited singlet state S_1_, the computed E_HB_ of O1–H2···N3 and O4–H5···N6 are 11.53 and 9.92 kcal/mol, respectively. Therefore, there are reasons to believe that both the two hydrogen bonds are strengthened in the S_1_ state. Moreover, hydrogen bond O1–H2···N3 should be strengthened more than O4–H5···N6 in the S_1_ state compared with the ground state (3.94 kcal/mol > 2.53 kcal/mol).

**Figure 4:**
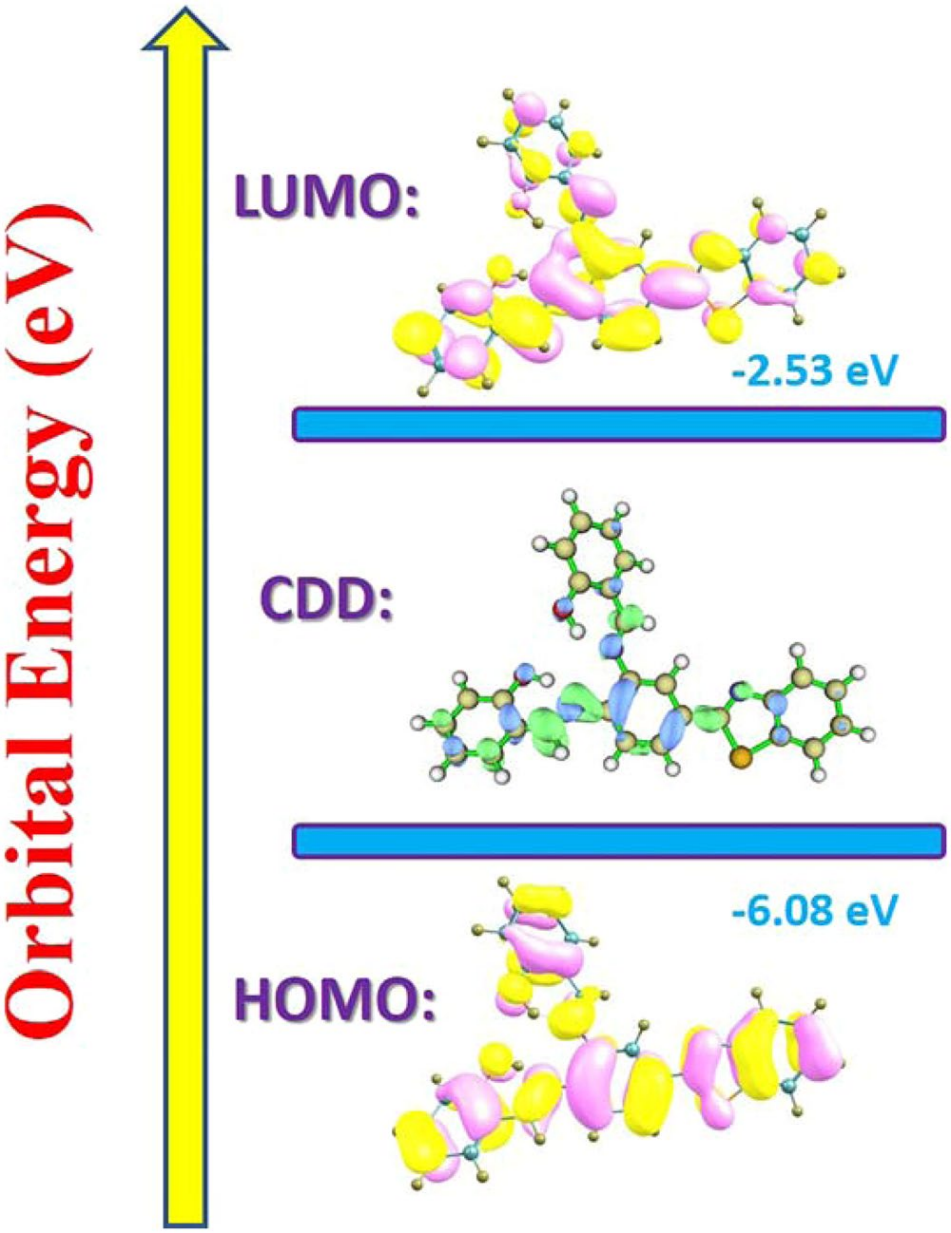
View of HOMO and LUMO orbitals for BTS system The CDD is also shown between HOMO and LUMO.

Since the changes of charge distribution resulting from photo-excitation process might result in different excited-state dynamical tendencies, we have calculated the vertical excitation energies of the first six low-lying excited states for BTS structures in DCM solvent. The corresponding theoretical results have been provided in Table 2. Other four transitions are shown in Table S1, ESI†. Our calculated first absorption peak (i.e., S_0_ → S_1_) for BTS is about 358 nm, which is consistent with experimental result (346 nm)^40^. For transitions S_0_ → S_2_, S_0_ → S_3_ and S_0_ → S_4_, it should be noted that the corresponding oscillator strengths are so small that they could be ignored during the photo-excitation behaviors. In addition, frontier molecular orbitals (MOs) are also discussed to uncover the charge redistribution case for BTS system. In Fig. 4, only HOMO and LUMO orbitals are displayed, which are principally involved in the electronic transition S_0_ → S_1_. Obviously, the S_1_ state is a dominant ππ*-type transition from HOMO to LUMO. For the charge redistribution about the hydrogen-bonded parts, the hydroxyl moieties contribute largely to HOMO, while their contributions decrease in LUMO. Hence, it can be concluded that the S_1_ state undergoes the intramolecular charge transfer process and the changes of electron densities of the hydroxide radical moieties could significantly affect the strength of the two hydrogen bonds.

**Table 2:** The excitation transitions, absorption energies *λ* (nm), oscillator strengths (*f*), corresponding configurations and percentage (%) for BTS form in DCM solvent.

Transition	*λ* (nm)	*f*	Composition	CI (%)
S_0_ → S_1_	358	0.5518	H → L	96.41%
S_0_ → S_2_	315	0.0417	H-1 → L	89.78%

Quantificationally, through the transition from HOMO to LUMO, the contributions of O1 and O4 to the molecular orbitals drops from 4.31% and 3.94% to 2.86% and 2.64%, whereas those of N4 and N6 increases from 6.73% and 6.57% to 9.04% and 8.65%, respectively. The increased charge densities of N4 and N6 should enhance the strength of the two intramolecular hydrogen bonds in some extent, which is consistent to the above conclusion. To be more visually, Fig. 4 also shows the charge density difference (CDD) map between HOMO and LUMO orbitals. It indicates that the net electron densities shift from hydroxyl groups to atoms N3 and N6 upon photoexcitation of BTS from state S_0_ to state S_1_. Particularly, the CDD figure reveals more obvious charge redistributions via hydrogen bond O1–H2···N3, which provides the tendency for ESIPT reaction^26–35^. Therefore, we can infer that hydrogen bond O1–H2···N3 should play a more important role during the ESIPT process of BTS.

**Figure 5:**
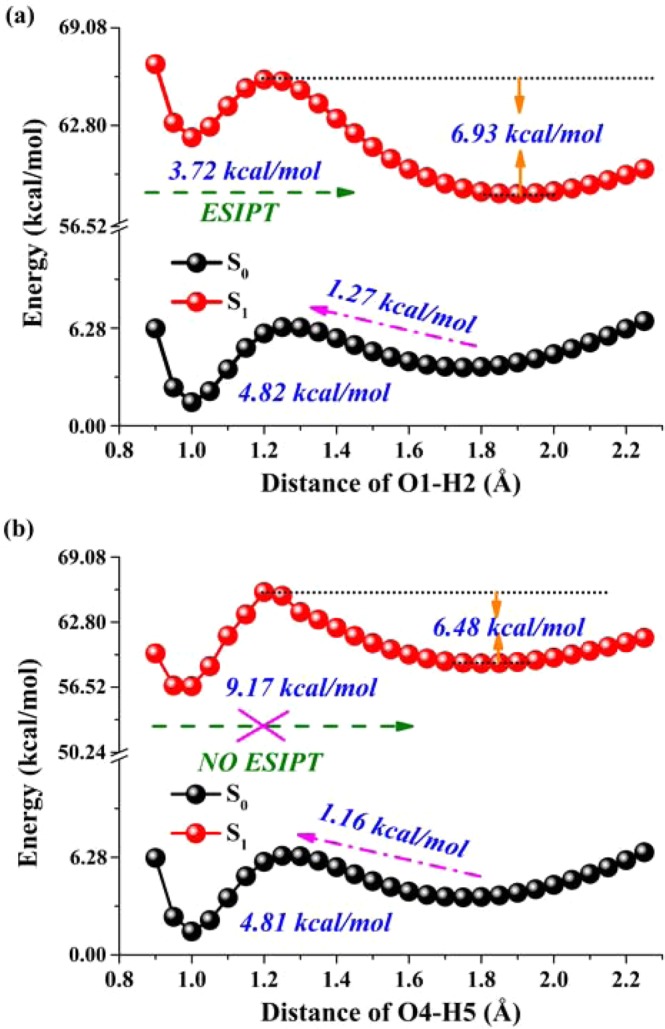
The constructed potential energy curves for BTS system along with hydrogen bond O1–H2···N3 (**a**) and O4–H5···N6 (**b**) based on fixing the O–H bond distance in both S_0_ and S_1_ states.

Constructing and analyzing potential energy curves should be an efficient manner to explore the ESIPT mechanism^26–35^. For BTS system, we could firstly exclude the excited-state double proton transfer process since the BTS-PT3 structure owns the highest energy (seen in Fig. 2). That is to say, among the four isomerides, BTS-PT3 is the most unstable one, which cannot be formed via ESPT process beginning from BTS structure. Therefore, we just consider the excited-state intramolecular single proton transfer case. Since BTS owns too hydrogen bonds, it is important to clarify along which hydrogen bond BTS undergoes the ESIPT process, which is not demonstrated in previous work^40^. Thus, the potential energy curves have been scanned considering two different situations on the base of constrained optimizations in both S_0_ and S_1_ states (see Fig. 5)^66–69^. Herein, one thing worth mentioning is that Duarte and co-workers have scanned the potential energy curves via relaxed optimizations in previous work^40^. However, their simulation result (barrierless ESIPT) is questionable since two emission peaks have been observed in experimental phenomenon^40^. In view of the relaxed scans way adopted by Duarte and co-workers, it might cross potential barrier due to the roughness of the method.

By constructing potential energy curves with constraining optimization method, we have reconsidered the ESIPT process of BTS. In Fig. 5(a), we investigated the ESIPT possibility via hydrogen bond O1–H2···N3. We find an energy barrier (3.72 kcal/mol) in transferring the proton (H2^+^) from O1 to N3 to form BTS-PT1. Then a barrier (9.17 kcal/mol) is found when transferring proton H5^+^ from BTS to BTS-PT2 in Fig. 5(b). Compared with Fig. 5(a), it can be infered that the ESIPT process via hydrogen bond O4–H5···N6 with an energy barrier 9.17 kcal/mol could be excluded. In addition, in view of the back PT behavior in the S_1_ state in Fig. 5(a), the reversed PT barrier from BTS-PT1 to BTS is 6.93 kcal/mol, which is higher than 3.72 kcal/mol. In other words, the backward PT process is thermodynamically unfavorable. Therefore, we could conclude the ESIPT process for BTS system as follows: upon photoexcitation to the S_1_ state, only one proton (H2^+^) can be transferred via the strengthened intramolecular hydrogen bond O1–H2···N3 due to the lowest potential energy barrier (3.72 kcal/mol) to form the BTS-PT1 configuration. Then it emits fluorescence and undergoes the reversed proton transfer back to the initial BTS form.

**Figure 6:**
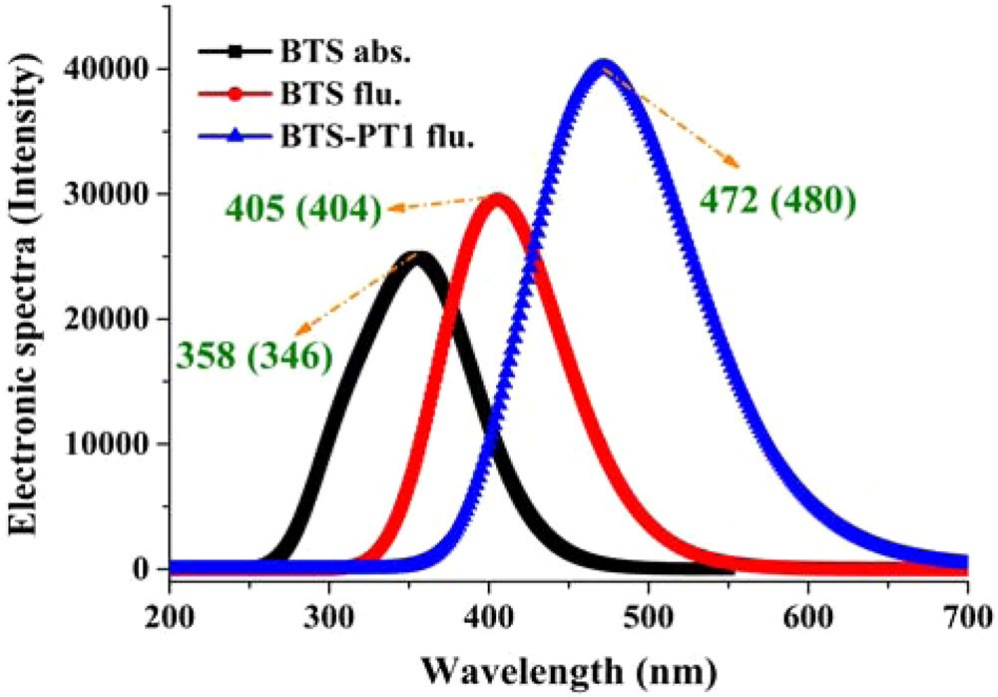
The simulated UV-Vis spectra for BTS system. The values in brackets stand for the previous experimental reports^40^.

The theoretical UV-Vis peaks of BTS and BTS-PTs are also simulated and compared with previous experimental results in Fig. 6. Upon photoexcitation, the computed absorption peak for BTS is about 358 nm, which is close to previous experimental spectra peak 346 nm^40^. After fast relaxation, the computed fluorescence peak of BTS is about 405 nm, which is also consistent to experimental spectra peak 404 nm^40^. Due to the ultrafast ESIPT process with low energy barrier 3.72 kcal/mol, the computed fluorescence peak 472 nm of BTS-PT1 is also in good agreement with experimental spectra peak 480 nm^40^. Therefore, the reliability of the theoretical methods of the present work is convincingly confirmed by the good agreements between calculated and experimental spectra peaks.

## Conclusion

The ESIPT process of BTS was studied with TDDFT method at the IEFPCM/B3LYP/TZVP theory level. Analyses about reduced density gradient (RDG) reveal the formation of two intramolecular hydrogen bonds in the ground state of BTS, which is similar to the results of ref. ^8^. Upon photoexcitation to S_1_ state, also similarly to the previous work, both of the two hydrogen bonds are strengthened, with hydrogen bonding energies increased by 3.94 kcal/mol and 2.53 kcal/mol respectively. The constructed potential energy curves along the two hydrogen bonds in S_1_ state verify that only proton H2^+^ can be transfered due to the relatively lower barrier along with O1–H2···N3 (3.72 kcal/mol) than O4–H5···N6 (9.17 kcal/mol), which is a little higher than the energy barrier 2.34 kcal/mol of transfering proton H5^+^ along with O4–H5···N6 in the previous work. This work not only presents a reasonable explanation for experimentally observed dual emission peaks (404 and 480 nm) of BTS system, but also clarifies the specific ESIPT mechanism.

## Supplementary information

Supplementary Information.
