# Supplementary material for: Theoretical insights into excited-state hydrogen bonding effects and intramolecular proton transfer (ESIPT) mechanism for BTS system
Source: Sci Rep. 2020 Mar 20;10:5119. doi: 10.1038/s41598-020-61804-7 (PMC7083891; doi:10.1038/s41598-020-61804-7)
Supplement: Supplementary file 1 — Supplementary Information. [file 41598_2020_61804_MOESM1_ESM.docx]

**Theoretical insights into excited-state hydrogen bonding effects and intramolecular proton transfer (ESIPT) mechanism for BTS system**

Jiemin Wang^1,2*^, Qiang Liu^1,2^, Dapeng Yang^3^

1. Department of Physics & Electronic Information, Luoyang Normal University, Luoyang 471934, P. R. China.

2. Henan Key laboratory of Electromagnetic Transformation and Detection, Luoyang 471934, P. R. China.

3. State Key Laboratory of Molecular Reaction Dynamics, Dalian Institute of Chemical Physics, Chinese Academy of Sciences, Dalian 116023, P. R. China

Corresponding author: Jiemin Wang, email: wangjiemin_1980@163.com.

**Table S1**. The excitation transitions, absorption energies *λ* (nm), oscillator strengths (*f*), corresponding configurations and percentage (%) for BTS form in DCM solvent.

| Transition | *λ* (nm) | *f* | Composition | CI (%) |
| --- | --- | --- | --- | --- |
| S_0_ → S_3_ | 302 | 0.0048 | H-2 → L | 81.92% |
| S_0_ → S_4_ | 295 | 0.0483 | H → L+1 | 69.62% |
| S_0_ → S_5_ | 285 | 0.00015 | H-1→ L | 67.24% |
| S_0_ → S_6_ | 254 | 0.00000 | H-4→ L+1 | 59.49% |


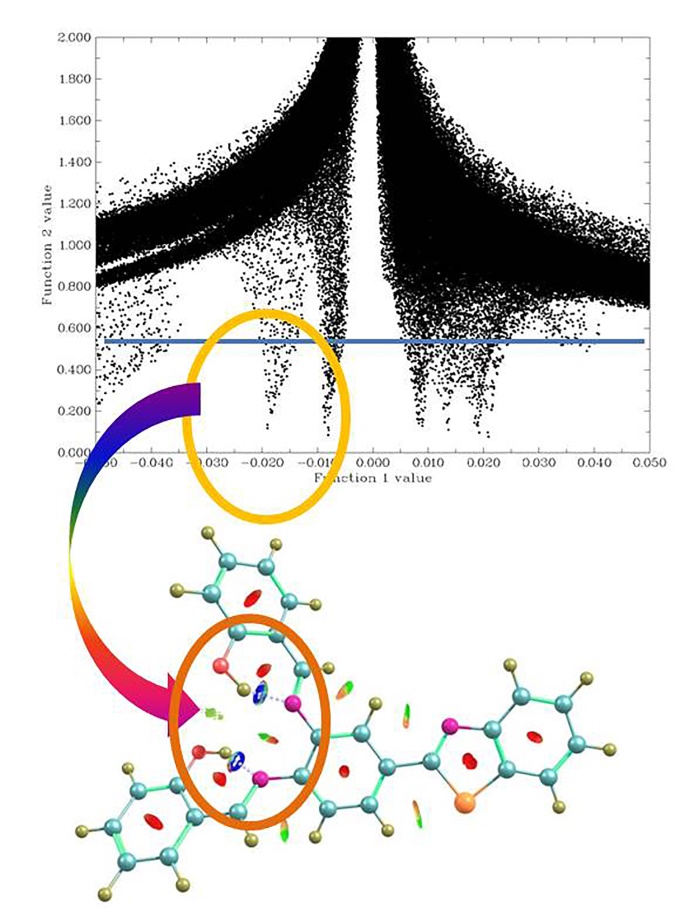


**Figure S1**. View of the RDG (Function 2) versus sign(*λ_2_*) *ρ* (Function 1) and the lower panel low-gradient (herein, s = 0.5 a.u.) isosurface for BTS structure in the S_0_ state. The weak interactions are shown below corresponding RDG (Red: steric effects; Green: VDW effects; Blue: hydrogen bonding effects).

In the process of investigating the possible correlation about weak interactions in real space, herein, we calculate and present the reduced density gradient (RDG) and sign(*λ_2_*)*ρ* as well as the lower gradient isosurface for BTS form in Figure S1. The contour value is set as 0.50 a.u., and the value range of RDG isosurface is set from -0.04 a.u. to 0.02 a.u. According to previous report by Johnson *et al.* [1], the multiple weak interactions could be revealed, namely, positive sign(*λ_2_*)*ρ* refers to steric effects, negative values of sign(*λ_2_*)*ρ* stand for hydrogen bonding interactions, and the values of sign(*λ_2_*)*ρ* near zero exhibit the van der Waals (VDW) effects. Obviously, the spikes located around -0.02 a.u. for BTS reveal the hydrogen bonding interactions for O1-H2···N3 and O4-H5···N6 in the ground state.

**References**

[1] Johnson, E., Keinan, S., Mori-Sanchez, P., Contreras-Garcia, J., Cohen, A., & Yang, W. Revealing noncovalent interactions. *J. Am. Chem. Soc*. **132,** 6498-6506 (2010).
